# Triglyceride-glucose index in the prediction of clinical outcomes after successful recanalization for coronary chronic total occlusions

**DOI:** 10.1186/s12933-023-02037-6

**Published:** 2023-11-08

**Authors:** Yu Yang, Mengqing Ma, Jian Zhang, Shiyu Jin, Dingxin Zhang, Xianhe Lin

**Affiliations:** Department of cardiology, The First Affiliated Hospital of Anhui Medical University, Hefei city, 230022 Anhui province China

**Keywords:** Chronic total occlusion, Triglyceride-glucose index, Insulin resistance, Percutaneous coronary intervention, Prognosis

## Abstract

**Background:**

Triglyceride-glucose index (TyG) has been widely used to predict cardiovascular outcomes. However, it remains unclear whether TyG holds prognostic significance for patients with coronary chronic total occlusions (CTO). Thus, our study aimed to evaluate the predictive accuracy and prognostic value of TyG in individuals who underwent successful percutaneous coronary intervention (PCI) for CTO.

**Methods:**

A total of 331 consecutive patients with ≥ 1 successful CTO-PCI were included. The baseline and angiographic data were acquired. The duration of follow-up ranged from 32 to 79 months, with a median of 44 months and an interquartile range of 39 to 67 months. The primary outcome measured was the occurrence of major adverse cardiac and cerebrovascular events (MACCE), including mortality, target vessel revascularization, recurrent myocardial infarction, and stroke.

**Results:**

After controlling for confounders, multivariate Cox regression analysis revealed that TyG remained statistically significant, regardless of being a continuous or categorical variable. In the partially adjusted regression model, the Hazard ratio (95%CI) for MACCE was 2.54 (1.12–5.79) in tertile 3 and 1.61 (1.22–2.12) per SD increase in the TyG.Kaplan-Meier survival analysis demonstrated significant differences in MACCE-free survival rates across tertiles of the TyG, as indicated by the log-rank test (p = 0.001). ROC analysis was conducted to evaluate the predictive ability of TyG for MACCE, resulting in an AUC of 0.677.

**Conclusion:**

The TyG index demonstrates independent predictive capabilities for MACCE in patients who have undergone successful CTO-PCI. These findings suggest that TyG holds the potential as a valuable tool in risk stratification and the identification of patients who may benefit from early intervention in the management of CTO.

**Supplementary Information:**

The online version contains supplementary material available at 10.1186/s12933-023-02037-6.

## Introduction

Coronary artery disease (CAD) is a global health burden, and within CAD, coronary chronic total occlusion (CTO) is observed in approximately 15–25% of patients undergoing coronary angiography [1, 2]. Despite the progression of percutaneous coronary intervention (PCI) technique, the prognosis of patients with CTO is often poor. Emerging evidence suggests that successful CTO-PCI is associated with several benefits compared to unsuccessful CTO-PCI and optimal medical treatment alone [3, 4]. These benefits include improvements in ventricular function, quality of life, and symptoms. However, the long-term prognosis of CTO-PCI remains a topic of debate, particularly for patients with metabolic abnormalities [2, 5, 6].

Metabolic abnormalities are important risk factors for CAD and cardiovascular adverse outcomes, in which insulin resistance (IR) plays a core role [7–9]. Although the homeostasis model assessment of insulin resistance (HOMA-IR) and the hyperinsulinemic-euglycemic clamp are established tools for evaluating IR, they are not commonly used in routine clinical practice. This is primarily due to several practical limitations, including complexity, time consumption, and cost. Triglyceride-glucose index (TyG) is a surrogate parameter that has been proposed as an indicator of IR. It is a simple calculation derived from fasting triglyceride (TG) and fasting blood glucose (FBG) levels [9, 10]. Considerable evidence suggests that TyG is superior to the HOMA-IR model in predicting arterial stiffness and metabolic syndrome [11, 12]. Moreover, TyG index has been shown to be associated with the incidence of cardiovascular diseases and their complications [13–15].

Previous studies have reported the association between TyG and adverse prognosis in different subgroups of CAD patients [15–17]. However, the prognostic values of TyG index have not been fully explored among patients with CTO,especially in successful CTO-PCI patients. As an important sub-type of CAD, it is urgently needed to investigate a valuable tool in risk stratification and the identification of CTO patients who may benefit from early intervention.Hence, the present study aimed to investigate the relationship between TyG index and prognostic outcomes in patients who have undergone successful PCI for CTO.

## Methods

### Study population

This retrospective cohort study was performed in compliance with the Declaration of Helsinki after obtaining approval from the Ethics Review Committee of the same university. From January 2016 to December 2019, 566 consecutive patients with ≥ 1 CTO lesion who received PCI treatment in the catheterization room were recruited. CTO lesions were characterized by total obstruction of the coronary artery, resulting in a complete absence of forward blood flow according to the thrombolysis in MI (TIMI) flow grade 0 criteria, with a duration of more than 3 months [18].

**Fig. 1:**
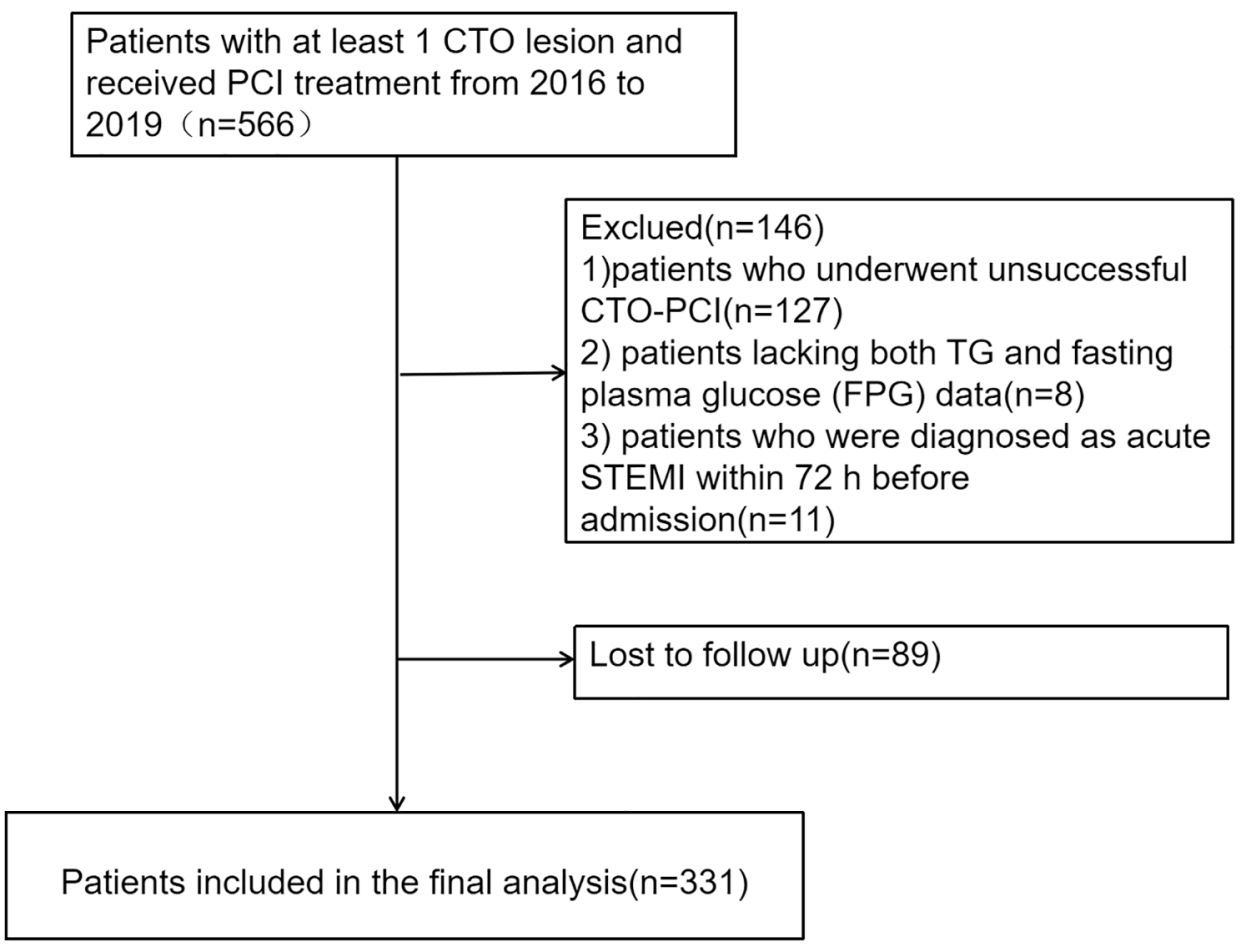
Flow diagram of this study

The following exclusion criteria were used: (1) patients with failed CTO-PCI (n = 127); (2) patients without triglyceride (TG) and fasting plasma glucose (FPG) data (n = 8); (3) patients diagnosed with acute ST-elevation MI within 72 h prior to admission (n = 11). Therefore, out of the initial 420 patients, a follow-up was conducted between July 2022 and August 2022 via telephone. A total of 331 patients (78.8%) successfully completed the follow-up (Fig. 1).

### Data collection and definitions

Demographic, clinical and angiographic information were acquired from medical records by trained clinicians who were unaware of the study objective. Peripheral venous blood was sampled before the coronary procedure in the morning following a minimum 8 h of fasting to determine blood biochemical parameters. TyG was measured as Ln [FPG (mg/dL) × fasting TG (mg/dL) / 2] [10]. SYNTAX scores were used to assess the severity of CAD. Multivessel disease was characterized by the presence of significant (> 50% diameter) stenosis in a minimum of two major coronary arteries. Poor coronary collateral circulation(CCC) (Grades 0 and 1) was grouped according to the Rentrop classification [19]. DM was evaluated based on the following criteria: FPG levels ≥ 7.0 mmol/L, random blood glucose (RBG) levels ≥ 11.1 mmol/L, 2-h plasma glucose levels after an oral glucose tolerance test (OGTT) ≥ 11.1 mmol/L, or the utilization of insulin or oral hypoglycemic agents [20]. Hyperlipidemia was characterized using the ICD-10 code E78 in combination with lipid-lowering medications or a total cholesterol level of ≥ 240 mg/dL [21]. Hypertension was described as having a systolic blood pressure of ≥ 140 mmHg or higher and/or a diastolic blood pressure of ≥ 90 mmHg, or being treated with antihypertensive drugs [22].

### PCI

The procedures for CTO-PCI were carried out by experienced cardiac intervention experts according to the current standard guidelines. Indications for coronary revascularization were based on symptomatic angina and/or positive results on functional ischemia studies.The success of the PCI procedure was determined based on the achievement of residual stenosis of less than 30% with TIMI flow grade 3 at the conclusion of the procedure,without any periprocedural complications.Following PCI, a standard dual-antiplatelet medication regimen was continued for a minimum of 12 months.The details of risk factor management after PCI were in accordance with current guidelines on myocardial revascularization [23].The treatment targets such as LDL-C and blood pressure are precisely managed according to each patient’s comorbidities and specific risk classification.The dosages of all medications were maximized to meet the recommendations as allowed by heart rate, blood pressure, and side effects in the absence of justifiable relative contraindications.

### Endpoints

MACCE occurrence was the primary endpoint of this study, which consisted of non-fatal stroke (hemorrhagic, ischemic, or unspecified), target vessel revascularization(TVR), non-fatal MI, and all-cause mortality (cardiac or non-cardiac mortality). The diagnosis of MI was defined in accordance with the criteria outlined in the Third Universal Definition [24]. TVR was specifically defined as the need for revascularization of the target vessel, which could be accomplished through PCI or coronary artery bypass grafting [25]. The information of target lesion revascularization (TLR) and any revascularization were also recorded. TLR has defned repeat revascularization at the same CTO lesion.Any revascularization was defined as any unplanned revascularization in follow up, including non-target vessels.

### Statistical analysis

Categorical data were presented with frequency or percentage, and were analyzed with the chi-square test or Fisher exact test accordingly. Continuous data were expressed as mean ± standard deviation (SD) or median (P25 and P75), as appropriate. In the case of a non-Gaussian distribution, the Kruskal-Wallis H test or Mann-Whitney U test was employed, whereas for a Gaussian distribution, the Student’s t-test or ANOVA test was used. Spearman or Pearson correlation analysis was conducted to investigate the relationships between TyG and cardiovascular risk factors. The survival curves were drawn by the Kaplan-Meier approach, and the differences between the two curves were compared by Log-rank. Multivariate Cox regression analysis was conducted to evaluate the independent correlation between TyG index and MACCE occurrence. Two regression models were constructed: model 1 (adjusted for gender and age), and model 2 (a partially adjusted model that included variables with p < 0.05 in univariate analysis). Subgroup analysis was performed based on gender, diabetes mellitus (DM), hypertension, hyperlipidemia, multi-CTO, and multivessel involvement to investigate potential differences in the relationship between TyG and MACCE among different subgroups. The p-value for interaction was determined to assess the significance of subgroup differences. Receiver operating characteristic (ROC) curves were obtained, and the area under the curve (AUC) was measured. Statistical analyses were conducted with SPSS v25.0 and R v4.1.3. Two-tailed P < 0.05 was deemed statistically significant.

## Results

### Baseline features

This study included 331 patients with successful CTO-PCI (mean age = 64.50 ± 10.36 years). All patients were assigned to three groups based on the tertile of TyG index level: TyG < 8.57 (tertile 1,n = 110); 8.57 ≤ TyG < 9.07 (tertile 2,n = 111) and TyG ≥ 9.07 (tertile 3,n = 110). Significant differences could be found in age, FPG, HDL-C, TC, TG, the proportion of DM, hypertension, hyperlipidemia, multiple CTO lesions, hypoglycemic drugs use, MACCE, and LCX lesion among the three groups. No significant difference was observed for the secondary endpoints and other indicators (Table 1).

**Table 1:** Baseline characteristics of the study population according to the tertiles of the TyG index

Variables	T1(n = 110)	T2(n = 111)	T3(n = 110)	p-value
TyG index	8.25 ± 0.27	8.82 ± 0.15	9.59 ± 0.41	< 0.001
General conditions				
Age(years)	66.46 ± 11.11	65.08 ± 9.68	61.95 ± 9.81	0.004
Male, n (%)	89(80.9)	74(66.7)	80(72.7)	0.056
BMI (kg/m2)	23.86 ± 3.43	24.35 ± 2.91	24.82 ± 2.52	0.059
LVEF (%)	55.40 ± 8.36	56.58 ± 8.29	56.68 ± 7.02	0.412
Risk factors, n (%)				
Current smoking	53(48.2)	55(49.5)	41(37.3)	0.133
Current drinking	36(32.7)	32(28.8)	32(29.1)	0.780
FH-CAD	4(3.6)	6(5.4)	4(3.6)	0.760
DM	19(17.3)	26(23.4)	60(54.5)	< 0.001
Hypertension	60(54.5)	77(69.4)	76(69.1)	0.032
Hyperlipidemia	44(40.0)	57(51.4)	88(80.0)	< 0.001
Prior stroke	29(26.4)	22(19.8)	27(24.5)	0.496
Prior PCI	28(25.5)	16(14.4)	16(14.5)	0.051
Prior MI	37(33.6)	31(27.9)	23(20.9)	0.106
Prior CABG	1(0.9)	2(1.8)	2(1.8)	0.804
Laboratory test				
FPG (mmol/L)	5.36 ± 0.95	5.95 ± 1.40	8.68 ± 4.26	< 0.001
TG (mmol/L)	0.95 ± 0.23	1.47 ± 0.31	2.50 ± 1.22	< 0.001
TC (mmol/L)	3.84 ± 1.11	4.11 ± 1.29	4.47 ± 1.24	0.001
LDL-C (mmol/L)	2.38 ± 1.01	2.53 ± 1.11	2.58 ± 1.15	0.368
HDL-C (mmol/L)	1.10 ± 0.27	1.05 ± 0.28	0.96 ± 0.22	< 0.001
eGFR (mL/min/1.73m2)	88.73 ± 22.21	90.33 ± 20.69	94.74 ± 20.75	0.100
UA (µmol/L)	350.87 ± 96.65	371.91 ± 106.21	343.39 ± 85.76	0.078
Cardiovascular medications, n (%)				
Aspirin	110(100.0)	108(97.3)	108(98.2)	0.118
Clopidogrel	84(76.4)	96(86.5)	88(80.0)	0.152
Ticagrelo	24(21.8)	14(12.6)	19(17.3)	0.194
Stains	109(99.1)	107(96.4)	110(100.0)	0.049
fibrates	7(6.4)	11(9.9)	17(15.5)	0.087
Beta-blockers	69(62.7)	74(66.7)	74(67.3)	0.743
CCB	26(23.6)	29(26.1)	27(24.5)	0.910
ACEI/ARB	51(46.4)	48(43.2)	47(42.7)	0.841
Hypoglycemic drugs	19(17.3)	26(23.4)	60(54.5)	< 0.001
Characteristics of CTO lesion				
Multiple CTO lesions	18(16.4)	6(5.4)	16(14.5)	0.028
Location of CTO lesions				
LAD	49(44.5)	46(41.4)	37(33.6)	0.235
LCX	24(21.8)	20(18.0)	37(33.6)	0.019
RCA	57(51.8)	54(48.6)	57(51.8)	0.862
Multivessel disease	92(83.6)	92(82.9)	93(84.5)	0945
SYNTAX score	20.27 ± 8.50	20.16 ± 7.21	20.17 ± 6.92	0.992
Poor CCC	59(53.6)	57(51.4)	60(54.5)	0.887
J-CTO score	0.75 ± 0.99	0.70 ± 0.82	0.78 ± 0.87	0.800
Treatment characteristics				
Number of stents for CTO-PCI				
1	45(40.9)	42(37.8)	47(42.7)	0.756
2	34(30.9)	49(44.1)	37(33.6)	0.097
≥ 3	30(27.3)	20 (18.0)	26(23.6)	0.257
Stent number	1.97 ± 1.10	1.92 ± 1.00	1.95 ± 1.10	0.930
Stent length (mm)	51.61 ± 32.28	49.23 ± 28.74	51.34 ± 33.95	0.829
Average diameter	2.87 ± 0.48	2.88 ± 0.33	2.88 ± 0.36	0.984
Outcomes, n (%)				
MACCE	9(8.2)	17(15.3)	27(24.5)	0.004
All-cause death	2(1.8)	6(5.4)	9(8.2)	0.100
Cardiovascular death	1(0.9)	2(1.8)	5(4.5)	0.195
MI	2(1.8)	2(1.8)	1(0.9)	0.804
TVR	4(3.6)	5(4.5)	11(10.0)	0.099
TLR	3(2.7)	5(4.5)	10(9.1)	0.100
Revascularization	6(5.5)	8(7.2)	14(12.7)	0.129
Stroke	1(0.9)	4(3.6)	6(5.5)	0.127

BMI, body mass index; LVEF, left ventricle ejection fraction;MI ,myocardial infarction;TyG index, triglyceride-glucose index; FH-CAD, family history of coronary artery disease;DM, diabetes mellitus; FPG, fasting plasma glucose;TC, total cholesterol;TG, triglyceride;LDL-C, low-density lipoprotein-cholesterol; HDL-C,high-density lipoprotein-cholesterol; eGFR,estimated glomerular filtration rate;UA, uric acid; ACEI, angiotensin-converting enzyme inhibitors, ARB angiotensin receptor blockers,TLR,target lesion revascularization;MACCE, major adverse cardiac and cerebrovascular events;Poor CCC,poor coronary collateral circulation

### Correlation between TyG index and cardiovascular risk factors

TyG was negatively correlated with age and HDL-C, while positively correlated with FPG, BMI, TG, TC, and eGFR (p < 0.05). No significant correlation was found between the TyG and LVEF, LDL-C, UA and SYNTAX score (Table 2).

**Table 2:** Correlations between the TyG index and cardiovascular risk factors

Variables	Correlation coefficient(r)	p-value
Age(years)	-0.215	< 0.001
BMI (kg/m2)	0.150	0.006
LVEF (%)	0.037	0.499
FPG (mmol/L)	0.576	< 0.001
TG (mmol/L)	0.867	< 0.001
TC (mmol/L)	0.267	< 0.001
LDL-C (mmol/L)	0.092	0.096
HDL-C (mmol/L)	-0.252	< 0.001
eGFR (mL/min/1.73m2)	0.164	0.003
UA (µmol/L)	-0.038	0.498
SYNTAX score	0.017	0.755

TyG index, triglyceride-glucose index; BMI ,body mass index; LVEF, left ventricle ejection fraction; FPG, fasting plasma glucose;TC, total cholesterol; TG, triglyceride; LDL-C, low-density lipoprotein-cholesterol; HDL-C, high-density lipoprotein-cholesterol; eGFR, estimated glomerular filtration rate; UA, uric acid

### TyG index and MACCE

**Fig. 2:**
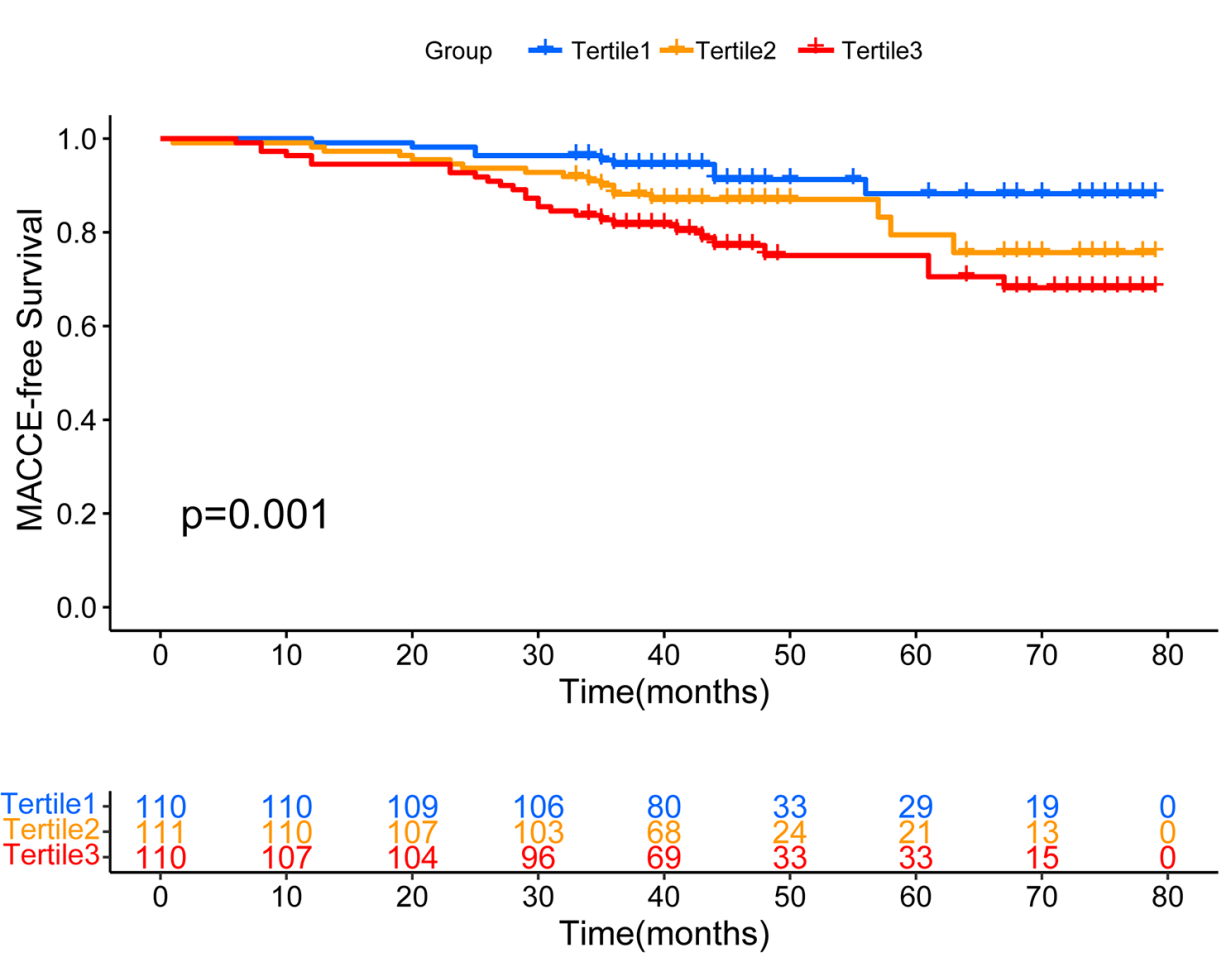
Kaplan–Meier curves for MACCE-free across the TyG tertile

All patients were followed-up for a period ranging from 32 to 79 months, with a median follow-up of 44 months and an interquartile range of 39 to 67 months. During this follow-up period, there were 53 cases of MACCEs, which accounted for 16.0% of the study population. These MACCEs consisted of 11 cases of non-fatal stroke (3.3%), 20 cases of coronary artery revascularization (6.0%), 5 cases of non-fatal MI (1.5%), and 17 cases of all-cause death (5.1%). The cumulative incidence of MACCE-free survival across tertiles of TyG was analyzed using Kaplan-Meier survival plots. The log-rank test demonstrated an obvious difference in the cumulative incidence of MACCE-free survival between the tertiles (p = 0.001), as depicted in Fig. 2. Furthermore, univariate Cox regression analysis was conducted to identify risk factors for MACCE. The analysis found that several factors were associated with an increased risk of MACCE, including DM, hypertension, FPG, TG, UA, use of hypoglycemic drugs, and TyG. Notably, the unadjusted HR (95%CI) for the probability of MACCE increased by one SD in TyG was 1.69 (1.33–2.15) (Supplementary Table 1).

After controlling for confounders, multivariate Cox regression analysis revealed that TyG remained statistically significant, regardless of being a continuous or categorical variable. In the partially adjusted regression model, the risk of MACCE elevated by 61% for each SD increase in TyG (HR = 1.61; 95%CI: 1.22–2.12). The partially adjusted HRs for MACCE in the middle and highest tertiles were 1.92 (95%CI: 0.84–4.40) and 2.54 (95%CI: 1.12–5.79), respectively, when compared to those in the lowest tertile. The results demonstrated a statistically significant trend of increased MACCE risk from tertile 1 to tertile 3 (p_trend_=0.026). (Table 3).

**Table 3:** Multivariable Cox regression analyses for the associations between TyG index and MACCE

TyG index	HR(95% CI)	
	Model 1	Model 2
Per 1 Unit increase	2.33(1.57–3.44)**	2.16(1.39–3.37)*
Per SD increase	1.69(1.33–2.15)**	1.61(1.22–2.12)*
Tertile1	1(Reference)	1(Reference)
Tertile2	2.16(0.95–4.88)	1.92(0.84–4.40)
Tertile3	3.42(1.58–7.40)*	2.54(1.12–5.79)*
P for trend	0.001	0.026

Model 1: adjusted for age and gender

Model 2: adjusted for variables with p-value < 0.05 in univariate analysis, including DM, hypertension, UA, LCX, average diameter and hypoglycemic drugs

MACCE, major adverse cardiac and cerebrovascular events; TyG index, triglyceride-glucose index; HR, hazard ratio; CI, confidence interval; SD, standard deviation

* p < 0.05

** p < 0.001

### The prognostic performance of TyG index

**Fig. 3:**
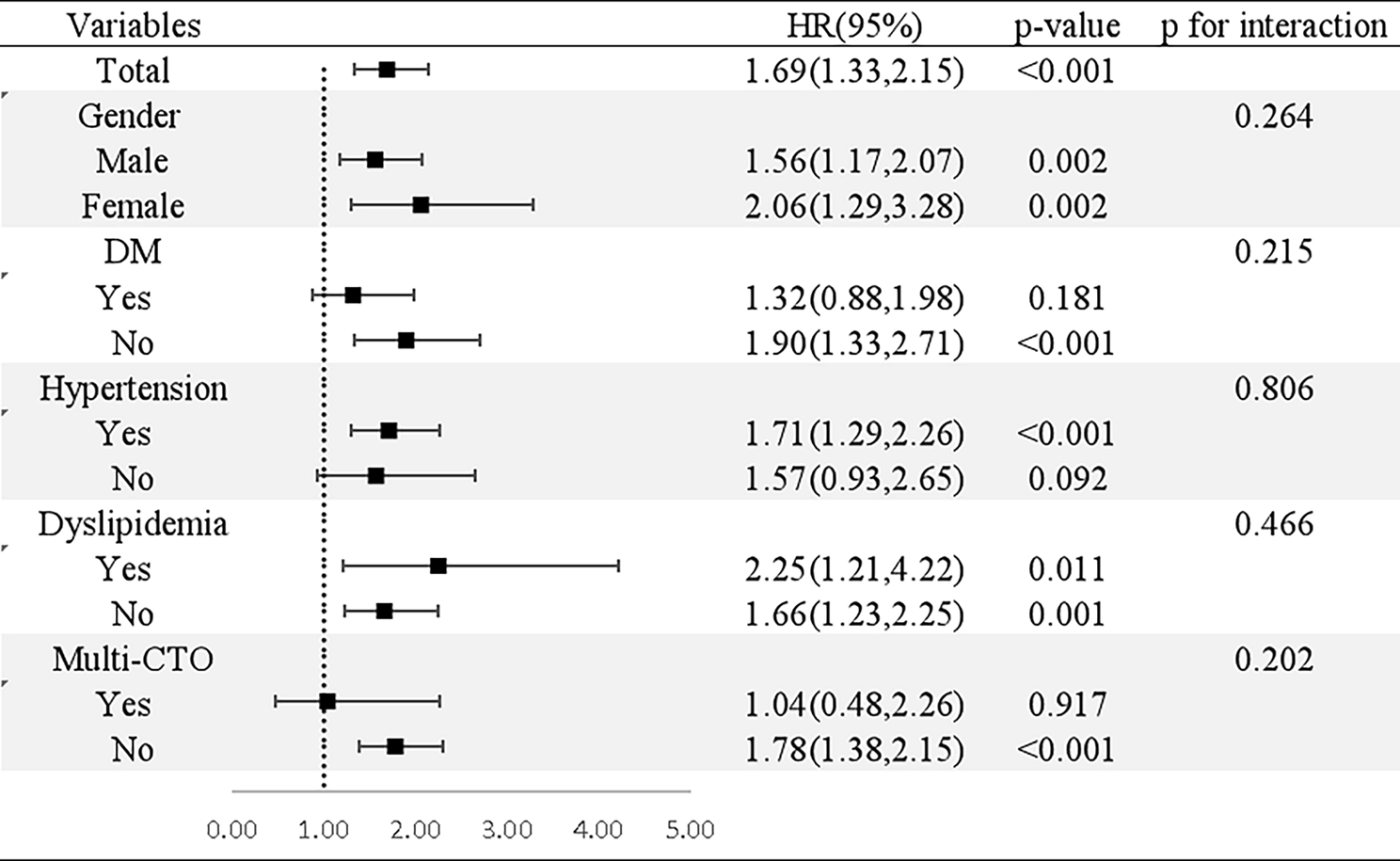
Subgroup and interaction analyses between TyG and MACCE in different subgroups

**Fig. 4:**
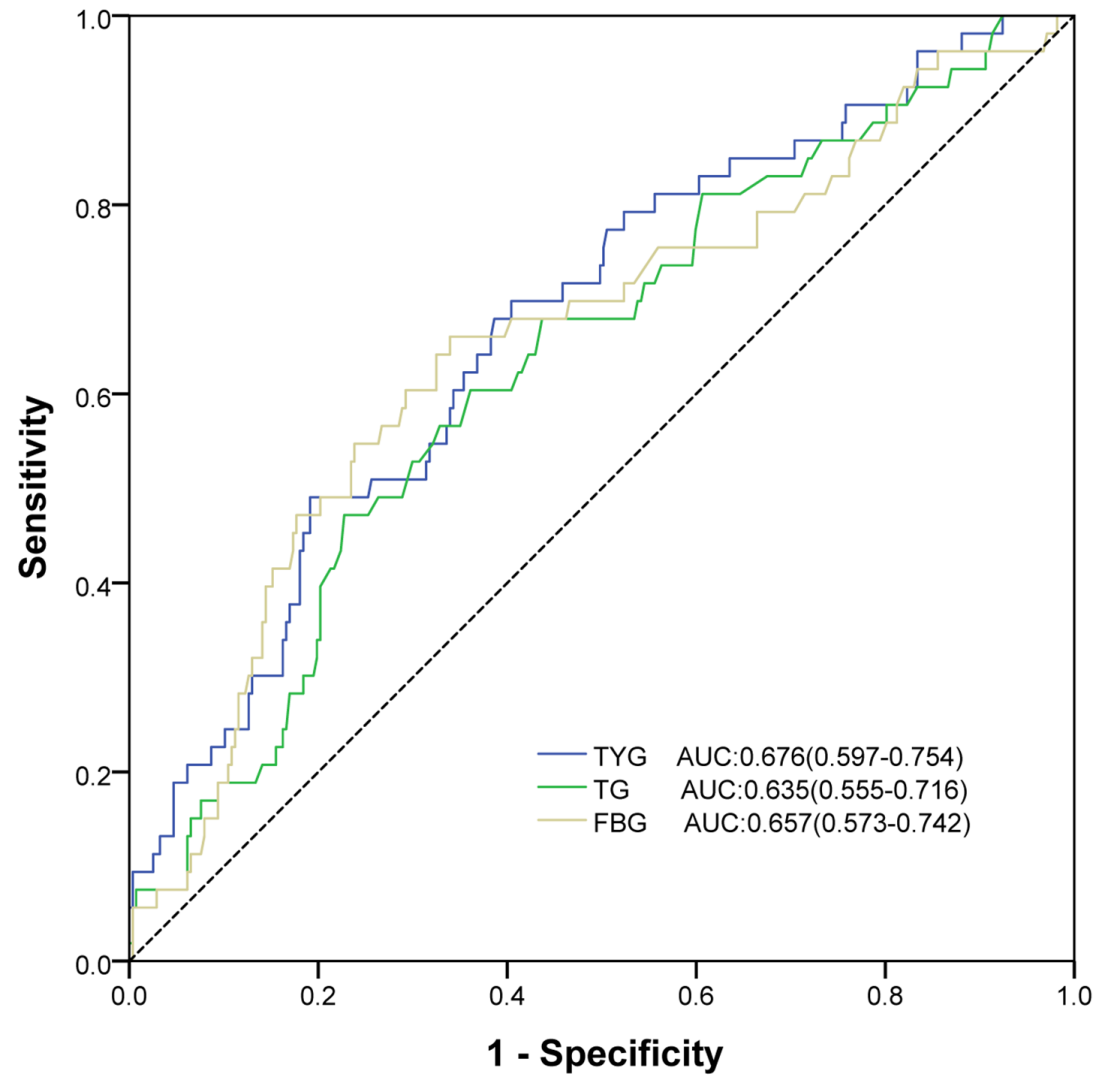
ROC curves of TyG index, TG, and FBG for predicting MACCE.

Subgroup analyses were conducted to examine the association between TyG and MACCE in specific subgroups of the study population. While no interaction was detected between TyG and gender, hypertension, DM, dyslipidemia, and multi-CTO for the incidence of MACCE (all p ≥ 0.202), a substantial connection between TyG and MACCE was found primarily among single CTO, patients with hypertension and patients without DM (Fig. 3). ROC analyses were carried out to further analyze the prognostic performance of TyG, yielding an AUC of 0.677 (95% CI 0.597–0.754)(Fig. 4). The AUC value of triglyceride and glucose levels were also calculated (Fig. 4).The interaction analysis of blood triglyceride and glucose in a COX regression for MACE found no interaction between them(p = 0.407).After analyze these points,the results show that TyG is more effective than triglyceride or blood glucose level alone.

## Discussion

To our knowledge, this is the first study that focuses on the relationship between TyG index and adverse outcomes in successful CTO-PCI patients. The following results were obtained: (1) TyG was markedly associated with traditional cardiovascular risk actors. (2) TyG was independently related to increased risk of MACCE in successful CTO-PCI patients. (3) The significant relationship between TyG index and MACCE was primarily found among single CTO, patients with hypertension, and patients without DM. Overall, TyG index proves to be a predictive indicator for patients after successful recanalization of CTO.

Despite the advancements in interventional devices and techniques for treating CTO, patients still face a significant risk of cardio-cerebrovascular events. Existing research has demonstrated that patients with CTO experience poorer prognosis compared to those without CTO [26]. In addition, metabolic abnormalities are important risk factors for the increased adverse prognosis after PCI equivalent of CTO [27]. As a core of metabolic abnormalities, IR contributes to cardiovascular disease by causing endothelial dysfunction, oxidative stress, and systemic inflammation [28–30]. In this context, TyG, a straightforward surrogate marker for IR, has been established as a prognostic indicator for CAD [31, 32]. Moreover, emerging studies have demonstrated a potential association between TyG and cardiovascular events in patients undergoing PCI [33, 34]. Zhu et al. reported that TyG was related to in-stent restenosis in ACS patients who underwent PCI [35]. Jiao et al. identified TyG as an independent predictor of long-term adverse outcomes in older ACS patients [16].Previous studies have also explored the role of TyG in predicting adverse outcomes in patients with CTO [36, 37]. However, after subgroup analysis, it was found that the prognosis of TyG for successful or failed CTO-PCI is still controversial. Therefore,the present study was dedicated to uncover the prognostic value of TyG in successfully treated CTO-PCI patients.

The exact mechanism underlying the association between TyG index and cardiovascular disease prognosis is remain unclear. The TyG index is a reliable marker of IR, which may contribute to the association.IR causes imbalanced glucose metabolism and chronic hyperglycemia, resulting in oxidative stress and inflammation that contributes to atherosclerosis and plaque progression [38].Moreover, IR can cause oxidative stress,endothelial disruption, systemic inflammation, clotting imbalance, poor myocardial reperfusion, and adverse vascular remodeling, all of which can compromise cardiac system [14, 38–40].IR, was also strongly associated with the prevalence and progression of coronary artery calcification ,which could be another important mechanism [41].The present study revealed significant associations between TyG and other risk factors. Previous research has also identified connections between TyG and renal insufficiency, dyslipidemia, and obesity [15, 42]. These correlations may partially explain the prediction of TyG on cardiovascular events.Taken together, these findings provide a plausible explanation for why a high TyG could predict cardiovascular events in patients with CTO.

Similarly, the association between TyG index and MACCE was found to be significant only in non-diabetic patients [32]. In patients with DM, accurately determining TyG becomes challenging due to the use of hypoglycemic agents. Moreover, in patients with DM, classical risk factors are the main predictors of cardiovascular events, and the role of IR alone is relatively weak [43]. Interestingly, our study revealed a significant association between TyG and MACCE primarily in patients with hypertension, which contradicts previous findings [32]. This discrepancy may be attributed to differences in data characteristics. Both TyG and IR can be influenced by different antihypertensive drugs [44]. Moreover, epidemiological studies have documented that IR predispose to hypertension [45]. The limited predictive value of TyG in patients with multiple CTOs may be attributed to the fact that these cases involve advanced coronary lesions with a higher burden and often coincide with more severe risk factors. These factors potentially explain some of the inconsistencies in the predictive power of TyG observed in this study.

As an indicator of glucose metabolism, TyG has been widely studied for the value in predicting the risk of CAD and evaluating prognosis [16, 33, 34].Notably,other indices of impaired glucose metabolism such as stress-induced hyperglycemia may hold prognostic value in patients with CTO, given their established association with adverse angiographic outcomes in patients with AMI [37, 46, 47].Further research could be designed to explore these indices to identify valuable tool in risk stratification and the identification of patients who may benefit from early intervention in the management of CTO.

Nevertheless, the present study has several limitations. Firstly, the sample size was limited due to the retrospective design conducted at a single center.Number of cardiovascular events which occurred during follow-up was also small,which might limit a sound statistical analysis and made it difficult to elaborate the associations between the TyG index and the individual components of MACCE.The results of stratified analysis for such a small number of participants or events need to be interpreted with caution. Secondly, despite performing multivariate analysis, there may still be unadjusted confounders that could impact the observed associations. Thirdly, the laboratory parameters were only assessed once at admission, and longitudinal data during follow-up were not systematically collected. Lastly, this study did not account for nutritional status and lifestyle factors, which could potentially influence TyG and outcomes. To strengthen the conclusions drawn from this research, further prospective multi-center studies with larger sample sizes are needed.

## Conclusion

In summary, TyG index could potentially be a predictor of MACCE in patients who undergo successful PCI for CTO. This finding highlights the significance of TyG as a simple and robust tool for predicting prognosis in CTO patients. By incorporating TyG index into clinical practice, healthcare professionals could potentially identify high-risk patients and implement appropriate measures to improve outcomes in this population.

### Electronic supplementary material

Below is the link to the electronic supplementary material.

Supplementary Material 1

## Data Availability

The datasets used and/or analyzed during the current study are available from the corresponding authors on reasonable request.

## List of Abbreviations

UA: Uric acid
HDL-C: High-density lipoprotein cholesterol
TyG: Triglyceride-glucose
CTO: Coronary chronic total occlusion
MACE: Major adverse cardiovascular event
TVR: Target vessel revascularization
ROC: Receiver operating characteristic
AUC: Area under the curves
CAD: Coronary artery disease
CVD: Cardiovascular disease
OR: Odds ratio
CI: Confdence interval
BMI: Body mass index
LVEF: Left ventricle ejection fraction
MI: Myocardial infarction
FH-CAD: Family history of coronary artery disease
DM: Diabetes mellitus
FPG: Fasting plasma glucose
TC: Total cholesterol
TG: Triglyceride
LDL-C: Low-density lipoprotein-cholesterol
eGFR: Estimated glomerular filtration rate
CCC: Coronary collateral circulation
