# Supplementary material for: Triglyceride-glucose index in the prediction of clinical outcomes after successful recanalization for coronary chronic total occlusions
Source: Cardiovasc Diabetol. 2023 Nov 8;22:304. doi: 10.1186/s12933-023-02037-6 (PMC10634094; doi:10.1186/s12933-023-02037-6)
Supplement: Supplementary file 1 — Supplementary Material 1 [file 12933_2023_2037_MOESM1_ESM.doc]

Supplementary Table 1 Univariate Cox regression analyses for MACCE

| Variables | HR | 95%CI | p-value |
| --- | --- | --- | --- |
| General conditions |  |  |  |
| Age(years) | 1.00 | （0.98,1.03） | 0.916 |
| Male, n (%) | 0.93 | （0.50,1.73） | 0.809 |
| BMI (kg/m2) | 1.06 | （0.96,1.16） | 0.252 |
| LVEF (%) | 0.99 | （0.96,1.02） | 0.558 |
| Risk factors, n (%) |  |  |  |
| Current smoking | 1.58 | （0.91,2.72） | 0.103 |
| Current drinking | 1.46 | （0.83,2.57） | 0.186 |
| FH-CAD | 0.40 | （0.06,2.91） | 0.368 |
| DM | 1.99 | （1.16，3.42） | 0.013 |
| Hypertension | 2.68 | （1.35,5.33） | 0.005 |
| Hyperlipidemia | 1.35 | （0.76,2.67） | 0.309 |
| Prior stroke | 1.25 | （0.66,2.34） | 0.496 |
| Prior PCI | 1.43 | （0.76,2.67） | 0.266 |
| Prior MI | 1.31 | （0.73,2.32） | 0.366 |
| Prior CABG | 1.28 | （0.18,9.24） | 0.809 |
| Laboratory test |  |  |  |
| FPG (mmol/L) | 1.09 | （1.03,1.14） | 0.002 |
| TG (mmol/L) | 1.33 | （1.11,1.60） | 0.002 |
| TyG index | 2.33 | （1.57,3.44） | <0.001 |
| TyG index（Per SD） | 1.69 | (1.33,2.15) | <0.001 |
| TC (mmol/L) | 0.98 | （0.78,1.22） | 0.837 |
| LDL-C (mmol/L) | 0.89 | （0.68,1.16） | 0.390 |
| HDL-C (mmol/L) | 0.24 | （0.08,0.73） | 0.012 |
| eGFR (mL/min/1.73m2) | 0.99 | （0.98,1.00） | 0.223 |
| UA (μmol/L) | 1.00 | （1.00,1.01） | 0.032 |
| Cardiovascular medications |  |  |  |
| Aspirin | 0.79 | （0.11,5.75） | 0.819 |
| Clopidogrel | 0.89 | （0.45,1.79） | 0.750 |
| Ticagrelo | 1.32 | （0.66,2.64） | 0.441 |
| Stains | 0.88 | （0.12,6.34） | 0.895 |
| Beta-blockers | 0.97 | （0.55,1.72） | 0.922 |
| CCB | 1.19 | （0.66,2.16） | 0.568 |
| ACEI/ARB | 0.67 | （0.38,1.17） | 0.155 |
| Hypoglycemic drugs | 1.99 | （1.16，3.42） | 0.013 |
| Characteristics of CTO lesion |  |  |  |
| Multiple CTO lesions | 0.94 | （0.40,2.19） | 0.876 |
| Location of CTO lesions |  |  |  |
| LAD | 1.380 | （0.771,2.470） | 0.279 |
| LCX | 2.31 | （1.33,0.777） | 0.005 |
| RCA | 0.71 | （0.41,1.22） | 0.210 |
| Multivessel disease | 1.54 | （0.66,3.60） | 0.319 |
| SYNTAX score | 1.02 | （0.98,1.05） | 0.416 |
| Poor CCC | 0.80 | （0.47,1.38） | 0.420 |
| J-CTO score | 1.08 | （0.81,1.44） | 0.593 |
| Treatment characteristics |  |  |  |
| Number of stents for CTO-PCI |  |  |  |
| 1 | 0.86 | （0.49,1.50） | 0.585 |
| 2 | 1.12 | （0.64,1.94） | 0.691 |
| ≥3 | 1.08 | （0.58,2.02） | 0.814 |
| Stent number | 0.99 | （0.77,1.27） | 0.936 |
| Stent length (mm) | 1.00 | （0.99,1.01） | 0.748 |
| Average diameter | 0.55 | （0.32,0.95） | 0.033 |

BMI, body mass index; LVEF, left ventricle ejection fraction;MI ,myocardial infarction;TyG index, triglyceride-glucose index; FH-CAD, family history of coronary artery disease;DM, diabetes mellitus; FPG, fasting plasma glucose;TC, total cholesterol;TG, triglyceride;LDL-C, low-density lipoprotein-cholesterol; HDL-C,high-density lipoprotein-cholesterol; eGFR,estimated glomerular filtration rate;UA, uric acid; ACEI, angiotensin-converting enzyme inhibitors, ARB angiotensin receptor blockers, MACCE, major adverse cardiac and cerebrovascular events;Poor CCC,poor coronary collateral circulation.
